# Surgical Outcomes of Adult Chiari Malformation Type 1: Experience at a Tertiary Institute

**DOI:** 10.7759/cureus.17876

**Published:** 2021-09-10

**Authors:** Meliha Gündağ Papaker, Anas Abdallah, İrfan Çınar

**Affiliations:** 1 Department of Neurosurgery, Bezmialem Vakif University, Istanbul, TUR; 2 Department of Neurosurgery, Aile Hospital, Istanbul, TUR

**Keywords:** surgical treatment, syrinx, posterior fossa decompression and duraplasty, magnetic resonance imaging, chiari malformation type i

## Abstract

Background

Chiari type I malformation (CM1) is a herniation of the caudal cerebellum and/or medulla oblongata into the upper spinal canal, occurring in pediatric and adult populations. We aimed to analyze the surgical outcomes of adult patients with CM1 consecutively treated with a posterior fossa decompression and duraplasty (PFDD) in a tertiary institution.

Patients and methods

We retrospectively reviewed the medical charts of 45 adult patients with CM1 who underwent PFDD at the Neurosurgery Department of our institution between January 2012 and December 2017. Radiological evaluation was based on pre- and postoperative syrinx/cord ratio, syrinx length, and regression of the ectopic cerebellar tonsils on coronal and sagittal magnetic resonance imaging (MRI) images, and clinical assessment of the patients was performed with the Chicago Chiari Outcome Scale (CCOS).

Results

Of the 45 patients included in the study, 25 (four men, 21 women) were diagnosed with symptomatic CM1 with an age average of 36.6±11.4 (18-66) years. Headache was the most common symptom (72.0%), while pinprick losses were prevalent in 13 (52.0%) patients. The mean postoperative CCOS score was 14.7±2.0 (8-16). Symptoms improved in 20 patients (80.0%) at the last follow-up. The mean regression in ectopic tonsils on midsagittal and coronal images were 9.1±1.8 (range: 0-16.5) mm and 8.3±1.2 (0-12.0) mm, respectively (p<0.05). The syrinxes had regressed completely or significantly in 7 (87.5%) of eight patients with syrinx.

Conclusion

Our findings showed that PFDD is sufficient to relieve most of the major symptoms and resolve the syrinx cavity without additional surgical interventions. The CCOS keeps its measurability of assessment of the clinical outcomes. A reliable radiological evaluation should be performed on midsagittal and coronal MRI images.

## Introduction

Chiari malformation type I (CM1) can be diagnosed incidentally with magnetic resonance imaging (MRI). CM1 is a congenital deformity characterized by a herniation of the cerebellar tonsillar below the foramen magnum level through the spinal column [1]. Due to the posterior fossa congestion, the clinical symptoms range from asymptomatic presentation to severe symptoms such as cognitive impairment and sleep apnea [2,3]. In the literature, several mechanisms were suggested to explain the pathogenesis of a CM1 as follows: 1) excess tissue in the posterior fossa by a tumor; 2) hemodynamic disturbances that increase intracranial pressure; 3) overcrowding caused by underdevelopment of the posterior fossa bony structures; and 4) downward movement of the central nervous system by events that lower intrathecal pressure [4]. While there is no consensus regarding the mechanism to identify the association between syrinx cavity and CM1, it is commonly assumed that this can be attributed to the obstruction at the level below the fourth ventricle in the subarachnoid space can impair the flow of the cerebrospinal fluid (CSF) in the central canal and the compression at the foramen magnum level that prevents it at the craniocervical junction [1,4-6]. However, the syrinx cavity does not appear in all patients with CM1.

In the management of patients with CM1, the role of duraplasty in relieving all presenting symptoms is unclear. Posterior fossa decompression (PFD) with suboccipital craniectomy is the gold standard surgical procedure. Some neurosurgeons argued that PFD without duraplasty is adequate to regress the syrinx and improve most clinical symptoms [6]. Conversely, several studies recommended PFD with duraplasty (PFDD) for patients with CM1 to normalize the CSF flow at the craniovertebral junction [1,7].

Aliaga and colleagues presented in 2012 Chicago Chiari Outcome Scale (CCOS), a scoring system to evaluate the surgical outcomes in patients with CM1 [1,8,9]. In the present study, we used the CCOS to analyze the outcomes of 25 consecutive patients with CM1 who underwent PFDD in a tertiary institution.

## Materials and methods

Study criteria

This retrospective study was approved by the ethical committee of Bezmialem Vakif University (BVU) (approval number: 2019/265). We analyzed the medical charts of 45 consecutive patients with CM1 who received PFDD for CM1 within a six-year period (2012-2017). Study criteria included the following: 1) adult (>18 years old) patients who were diagnosed using coronal and sagittal MRI images after complaining of typical functional and neurological symptoms for CM1, 2) those who underwent PFDD (adequate posterior fossa decompression, posterior laminectomy of the C1 vertebra, and expansile duraplasty), 3) those who signed written consent for the surgical procedure and participation in the study, and 4) those who were followed up for a minimum of 3 years (attended all control visits and underwent pre-and postoperative coronal and sagittal MRI).

We excluded patients who had syndromic diseases, other cranial and spinal pathologies, or ventriculoperitoneal shunt for hydrocephalus, recurrent CM1 symptoms underwent PFD and those lost to follow-up. Finally, we analyzed 25 adult patients with CM1 (Table 1).

**Table 1:** The analyzed adult CM1 patients according to our study criteria (n = 25) pts: patients; w/: with; CM1: Chiari malformation type I; PTR: Physical therapy and rehabilitation; Pre: Preoperative; PO: Postoperative; No pre/PO adequate MRIs: If the patients lost comparative sagittal or coronal images of MRIs; PDF alone: There no duraplasty or/and C1 laminectomy was applied; additional pathologies: such as hydrocephalus, intracranial or spinal lesions.

Variables	The number of Pts
Study period	Jan 2012 – Dec 2017
All pts diagnosed w/ CM1	121
Asymptomatic pts	29 (24.0%)
Symptomatic pts	92 (76.0%)
Observed pts w/o treatment	15 (16.3%)
Conservative ± PTR	32 (34.8%)
Surgically treated	45 (48.9%)
Pediatric pts (< 18 years)	7 (15.6%)
Syndromic adult pts	2 (4.4%)
No pre/PO adequate MRIs	3 (6.7%)
Additional pathologies (+)	1 (2.2%)
Received PDF alone	2 (4.4%)
For recurrent CM1	2 (4.4%)
Lost follow-up	3 (6.7%)
Pts included in the study	25 (55.6%)

Radiological investigations

**Figure 1:**
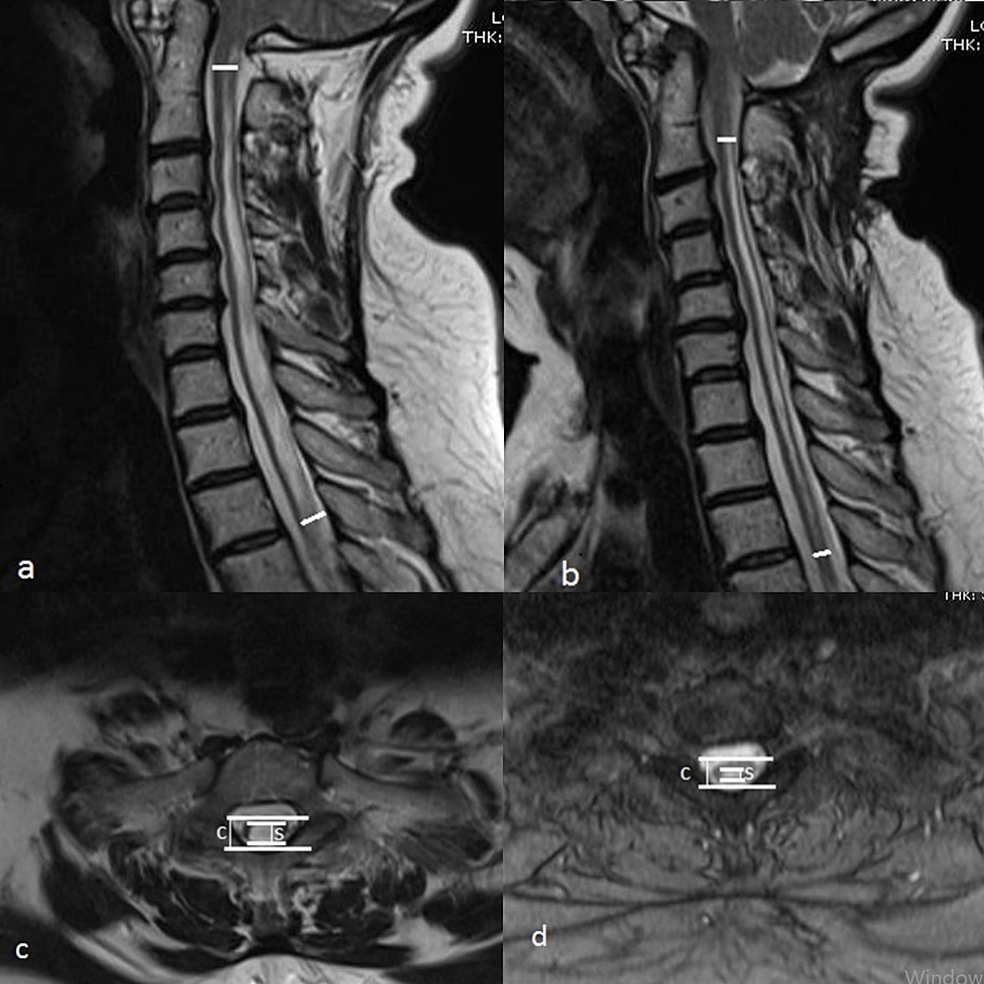
The pre- and postoperative MRIs of a 50-year-old female patient with CM1. The pre- and postoperative MRIs of a 50-year-old female patient with CM1: (a) Preoperative MRI demonstrated a syrinx extended from the C2 to T3 vertebrae level (b) The sixth postoperative month MRI showed no significant regression of the syrinx’ length; however, the patient’s presenting symptoms improved related to the regression in the S/C ratio (c) Preoperative axial MRI showed S/C ratio (d) Postoperative sixth MRI showed a regressed S/C ratio. S/C ratio: syrinx/cord ratio; CM1: Chiari malformation type I

**Figure 2:**
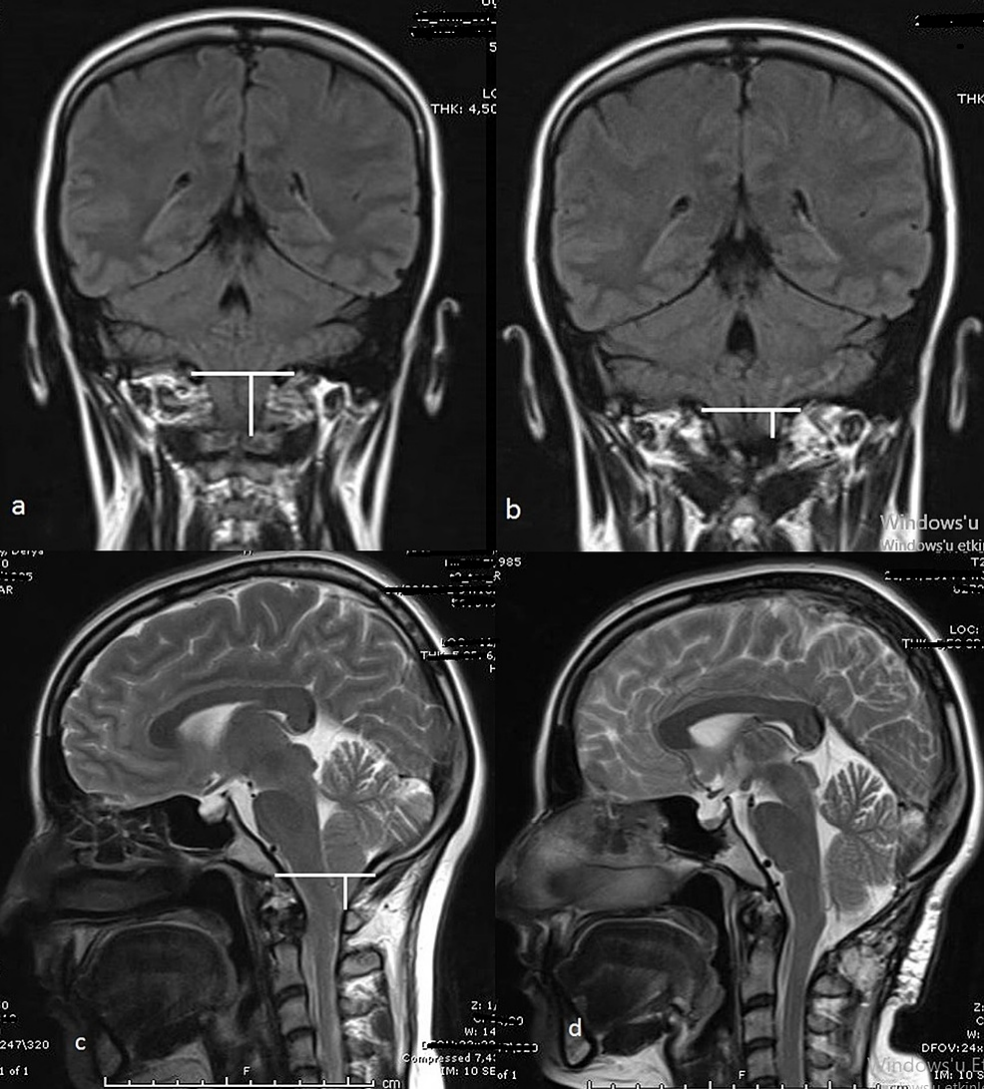
The pre- and postoperative MRIs of a 27-year-old female patient with CM1. The pre- and postoperative MRIs of a 27-year-old female patient with CM1: (a) Preoperative coronal image showed asymmetric cerebellar tonsils (the left herniated greater than the right side) (b) The 6th postoperative coronal image showed the regression of cerebellar tonsils (c) The preoperative sagittal image demonstrated the herniated cerebellar tonsils (d) The postoperative sagittal image showed the regression of the herniated tonsils. CM1: Chiari malformation type I

Sagittal, axial, and coronal images of pre-and postoperative MRIs were evaluated by two senior neuroradiologists who were blinded to the surgical results of the patients. In cases of different evaluations, the final decision was taken after a consensus was reached. In the first evaluation, an acceptable interobserver agreement was seen in 22 of 25 (88.0%) patients with Cohen’s Kappa of 0.80.5. The measurements were compared between pre-and postoperative MRIs: syrinx/cord ratio (S/C) based on the maximal anteroposterior diameters on axial images, maximal syrinx length on sagittal images (Figure 1), and regression of the herniated ectopic cerebellar tonsils measured by the descent from the estimated McRae’s line to the lowest level of tonsils into the upper spinal canal on the sagittal images and the descent between the horizontal plane from the foramen magnum and the lowest level of tonsils on the coronal images [1] (Figure 2).

Surgical approach

All patients underwent surgery in the same center (BVU) by more than one surgeon (AA and MGP) using the same surgical approach (PFDD). While in prone position under general anesthesia, the patient’s head was slightly flexed and fixed with pins. A midline incision was made from the inion to the upper cervical vertebra. After dissection of the paraspinal muscles, suboccipital craniectomy and C1 laminectomy were performed using bone rongeurs and/or a high-speed drill. The dura was incised in a Y-shape fashion under a white-lighted operative microscope. In the syrinx case, after entering and lateral clipping the arachnoid, the Magendie foramen was inspected by gently separating the tonsils and appreciating the place of the posterior inferior cerebral artery. In suspicion of the presence of the webs, adhesions, or obstacles, the fourth ventricle was exposed. Arachnoid adhesions were released by sharp dissection to facilitate the CSF flow. After hemostasis, a tight closure duraplasty was performed using an autograft or synthetic graft. We used Fibrin Sealant Products (TISSEEL®; Baxter International, Deerfield, USA). Multilayers were closed following their anatomical structure.

Postoperative assessment and follow-up

All patients were assessed postoperative days 1 and 14 and months 6, 12, and 24. For assessment of the clinical improvement at 6 months, the CCOS were recorded. To standardize the postoperative assessment of surgical outcome for patients with CM1, we used the CCOS that the score we believed it is a reliable and objective scoring system. The CCOS was firstly defined by Aliaga et al in 2012 [9] and several studies supported its reliability [1,8]. This scoring system quantifies specific aspects of outcomes after decompressive surgery in patients with CM1 to predict how well surgical intervention will treat a patient with a specific constellation of presenting complaints [9]. For radiological evaluation, at postoperative month 6, pre-and postoperative MRIs were compared to evaluate the regression on coronal and sagittal images. In the CM1-associated syrinx cases, the S/C ratio and length of the syrinx on sagittal MRI were compared pre-and postoperatively.

Statistical analysis

The data were expressed as frequency (percent), mean ± SD, and ranges. For evaluation of the differences between both groups, we utilized paired t-test, with p-values<0.05 that were considered significant statistically. All statistical analyses were performed by SPSS Statistics software (Version 21.0, IBM Corp, Armonk, USA). An independent samples t-test was used to compare pre- and postoperative radiological measurements.

## Results

A total of 45 patients were diagnosed with symptomatic CM1 at BVU from January 2012 to December 2017. Of them, 25 (21 women and four men) who underwent PFDD for CM1 have met the study criteria (Table 1). The mean age of the analyzed sample was 36.6±11.4 (range: 18-66) years. Headache was the most common symptom in 18 (72.0%) patients (Table 2), and pinprick loss in 13 (52.0%) patients (Table 3). 

**Table 2:** Baseline demographic and clinical characteristics of 25 patients pts: patients; F: Female; M: Male; CM1: Chiari malformation type I; Typical headache for CM1: occipital, Valsalva induced, tussive and exertional headache; Atypical headache: Poorly localized frontal, temporal, or nonspecific headache; Neuropathic pain: dysesthesia, paresthesia, and hyperesthesia; Dissociated sensory loss: loss of pinprick and temperature sensation; LOS: length of hospital stay; IP: Intraoperative.

Variables	The number of the pts (%)
Number of pts	25 (100%)
Sex (F/M)	21/4 (84.0%)
Mean age (years)	36.6±11.4 (18-66)
Presenting symptoms	
1) Pain and dysesthesia: 22 pts (88.0%)	
* Typical headache for CM1	14 (56.0%)
* Atypical headache	4 (16.0%)
* Neuropathic pain	12 (48.0%)
* Neck pain	9 (36.0%)
* Upper extremities pain	3 (12.0%)
* Spinal pain (back ± low back)	2 (8.0%)
* Chest pain	2 (8.0%)
2) Neurological non-pain: 17 pts (68.0%)	
* Dissociated sensory loss	13 (52.0%)
* Vertigo/dizziness	8 (32.0%)
* Ataxia	5 (20.0%)
* Dysphagia	4 (16.0%)
* Tinnitus	6 (24.0%)
* Disequilibrium	4 (16.0%)
* Paresis	3 (12.0%)
* Nystagmus	3 (12.0%)
* Impaired reflexes	4 (16.0%)
* Myelopathy	3 (12.0%)
* Fecal ± Urine incontinence	2 (8.0%)
3) Functional symptoms: 13 pts (52.0%)	
* Fatigue	11 (44.0%)
* Syncope	2 (8.0%)
* Dysarthria	1 (4.0%)
* Cognitive impairment	2 (8.0%)
* Hoarseness	1 (4.0%)
4) Sleep apnea category: 5 pts (20.0%)	
* Sleep apnea	3 (12.0%)
* Severe snoring	2 (8.0%)
* Recurrent aspiration	3 (12.0%)
* Hiccoughs	1 (4.0%)
Symptom duration (months)	16.4±9.6 (3-24)
Follow-up period (months)	58.5±22.8 (26-97)
Mean LOS (days)	3.6±1.0 (2-5)
Mean IP blood loss (ccs)	190.1±36.4 (50-250)

**Table 3:** The clinical findings of 25 patients pts: Patients; Headache w/ Valsalva: Valsalva induced, tussive and exertional headache; Neuropathic pain: dysesthesia, paresthesia, and hyperesthesia; Dissociated sensory loss: loss of pinprick and temperature sensation; DTRs: Deep tendon reflexes.

Clinical findings	The number of the pts (%)
Headache w/ Valsalva	14 (56.0%)
Pinprick loss	13 (52.0%)
Impairment of the sensation	12 (48.0%)
Vertigo test	8 (32.0%)
GAG reflex	6 (24.0%)
Impaired DTRs	8 (32.0%)
Positive Romberg sign	4 (16.0%)
Paresis	3 (12.0%)
Musculature atrophy	3 (12.0%)
Urine incontinence ± Sphincter laxity	2 (8.0%)

We detected intraoperatively six (24%) patients with arachnoid webs after exposing the fourth ventricle. The mean postoperative CCOS score was 14.7±2.0 (8-16). Symptoms improved in 20 patients (80.0%) at the last follow-up of an average of 78.4±21.6 (36-108) months (Table 4).

**Table 4:** Co-malformations** and the surgical outcomes pts: Patients; CCOS: Chicago Chiari Outcome Scale; Recovered: Relieved all main symptoms; Improved: Continuing at least one main symptom with obvious neurological improvement in other main symptoms; Unchanged: continuing all major symptoms without any improvement; Worse: Worsening at least one main symptom or appearing at least one new neurological deficit). ** The number of these patients was relatively small since we excluded the pathologies that required additional surgeries for different pathologies. The pathologies in these patients were mild.

Variables	The number of the pts (%)
Mild scoliosis	4 (16.0%)
Mild kyphosis	1 (4.0%)
Mean CCOS value	14.7±2.0 (8-16)
Outcomes	
Recovered	20 (80.0%)
Improved	3 (12.0%)
Unchanged	2 (8.0%)
Worse	0

Tonsillar ectopia was detected in 17 of 25 patients, with a mean regression and pre-and postoperative measurement of 8.3±1.2 (range: 0-12.0) mm, 14.2±2.7 (4.0-17.0) mm, and 5.9±0.9 (0-7.9), respectively (p<0.001), based on coronal MRI and 9.1±1.8 (0-16.5) mm, 13.4±3.6 (3.0-18.0) mm, and 4.3±1.8 (1.2-12.0), respectively (p<0.001), based on midsagittal images. The syrinxes had regressed completely or significantly in 7 (87.5%) of eight patients with syrinx (Table 5).

**Table 5:** Radiological parameters pts: patients; midsag.: Midsagittal; Pre: Preoperative; PO: Postoperative; max: maximum; S: Syrinx; C: spinal cord; S/C: the ratio of syrinx to the spinal cord on axial sequences; (+): presence; AP: Anteroposterior. * The number of the patients who had ectopia on coronal sequences (not all patients had ectopia on coronal sequences). ** These numbers were given for the only patients with persist syrinx, the patients with the regressed syrinxes were excluded).

Variables	The number of the pts (%) and mean values with ranges
Ectopia on midsag. images:	25 pts (34.7%)
Mean Pre tonsil hernia (mm)	13.4±3.6 (3-18)
Mean PO tonsil hernia (mm)	4.3±1.8 (1.2-12)
Mean regression (mm)	9.1±1.8 (0-16.5)
Unchanged pts (%)	2 (8.0%)
Ectopia on coronal images:	17 pts (32.1%)*
Mean Pre tonsil hernia (mm)	14.2±2.7 (4.0-17.0)
Mean PO tonsil hernia (mm)	5.9±0.9 (0-7.9)
Mean regression (mm)	8.3±1.2 (0-12.0)
Unchanged pts (%)	2 (8.0%)
Syrinx (+) pts (%): 8 (32.0%)	
Mean Pre axial S/C ratio	0.84±0.1 (0.63-0.92)
Mean Pre max AP diameter of S	5.1±2.4 (3.1-8.8)
Mean Pre max AP diameter of C	5.8±2.6 (3.6-9.6)
Mean PO axial S/C ratio**	0.55±0.2 (0.31-0.76)
Mean PO max AP diameter of S	2.2±1.0 (1.5-3.8)
Mean PO max AP diameter of C	4.4±0.6 (3.9-6.0)
Mean Pre length of syrinx	54.9±58.6 (11.3-160)
Mean PO length of syrinx	45.6±57.8 (0-160)
Fully regressed syrinx pts (%)	3 (37.5%)
Unchanged syrinx pts (%)	1 (12.5%)
Significantly regressed S pts (%)	4 (50%)
Syrinx required syringopleural shunt pts (%)	0

The comparison between the pre -and postoperative radiological measurements was shown in Table 6.

**Table 6:** Comparison between the pre-and postoperative radiological measurements P < 0.05: statistical significant; pts: patients; Pre: Preoperative; PO: Postoperative; max: maximum; S: Syrinx; C: spinal cord; S/C: the ratio of syrinx to the spinal cord on axial sequences.

Measurement	Mean	N	SD	t	p
Pre ectopia on midsagittal images (mm)	13.4	25	3.6	11.3	< 0.001
PO ectopia on midsagittal images (mm)	4.3	25	1.8		
Pre ectopia on coronal images (mm)	14.2	17	2.7	12.02	< 0.001
PO ectopia on coronal images (mm)	5.9	17	0.9		
Pre S/C ratio	0.84	8	0.1	3.52	0.005
PO S/C ratio	0.55	5	0.2		
Pre length of the syrinx (mm)	54.9	8	58.6	0.28	0.78
PO length of the syrinx (mm)	45.6	5	57.8		

Surgical complications are given in Table 7.

**Table 7:** Surgical complications pts: Patients; CSF: Cerebrospinal fluid; PO: Postoperative; SS: Surgical site; SSIs: surgical site infections.

Complication	The number of the pts (%)
CSF-related complications	2 (8.0%)
CSF fistula	0
Pseudomeningocele	1 (4.0%)
Chemical meningitis	1 (4.0%)
Delayed SS closure related to PO SSIs	1 (4.0%)
Unchanged neurological function	1 (4.0%)

## Discussion

CM1 is a congenital anomaly characterized by the herniation of the cerebellar tonsils or medulla oblongata through the foramen magnum down into the upper cervical spine [1]. An embryological origin of the disease was discussed; however, CM1 can also represent a secondary to disorders unrelated to the posterior cranial fossa hypoplasia [1,10]. Several studies have shown that PFD with or without duraplasty results in good outcomes for symptomatic CM1 with or without syrinx cavity [1,6]. However, in the presence of ventral compressions such as the clival masses or bone abnormalities such as basilar invagination, the treatment of the anterior craniovertebral decompression is mandatory to achieve good outcomes [1], which is possible by the exploration of the fourth ventricle followed by duraplasty after bony decompression and removal of the veils, pouches, or webs (the intradural membranes observed at the fourth ventricle outlet of the foramen of Magendie that are interfering with the CSF flow) [1,10]. One recently published study recommended a C1-C2 fixation as the surgical option of CM1 [11]. However, there is no consensus regarding the optimal treatment approach among all neurosurgeons works in different neurological centers worldwide. We believe that PFDD allows sufficient decompression to normalize the passage of the CSF flow at the craniocervical junction; therefore, in our study, we analyzed the patients who received PFDD only to understand the surgical outcomes for CM1.

The etiology of CM1 can affect the CSF hydrodynamics, resulting in clinical symptoms such as typical occipital tussive headaches, changes in cognitive and neuropsychological functions, myelopathy, and the involvement of the upper and the lower cranial nerves [1,10,11]. Obstruction of the fourth ventricular level can alter the craniospinal pressure that has been advocated in the genesis of the syrinx cavity. However, the exact relationship between the syrinx and arachnoid webs is not yet completely described [5,10] The selection criteria for surgery remain the issue in patients with CM1 as this should depend mainly on the objective measurements such as the degree of CSF flow obstruction rather than subjective or atypical radiological findings such as the herniation degree and nonspecific symptoms. According to some recently published studies, the CSF flow can be a better alternative to assess the surgical candidate, evaluate surgical outcomes, and follow-up the treated patients with CM1 [1,12,13]

The CCOS is a measurable clinical scale and is still preferable in conducting a clinical evaluation of patients with CM1 patients in several institutions worldwide. The scale was Introduced by Aliaga et al., the scale indicates good, unchanged, and worsen surgical outcomes with 101, 39, and 6 points, respectively [9]. In another study, the same authors calculated the CCOS as 13-14 points in 112, 9-12 points in 48, and 4-8 points in 7 of 167 patients [8]. The mean CCOS score varies among studies: Yarbrough et al. found it at 14 points in a study of 292 patients [14], while Papaker et al at 14.1 in carefully selected 72 adult patients with CM1 [1]. Lee et al. divided the patients with CM1 into two groups based on the procedure: PFDD (n=36) and PFD (n=29) groups. They found the mean CCOS as 14.6 and 14.7, respectively [15]. Our findings were in line with these studies.

Several studies have evaluated radiological findings postoperatively [1,16]. In their study of 87 patients with CM1, Xie et al. found the mean pre-and postoperative S/C ratio of 0.66 and 0.42, respectively [16], and the mean pre-and postoperative syrinx lengths were 10.91 and 5.52 mm, respectively. The authors reported the syrinx resolution was observed in 78 (91.8%) of 87 patients [16]. In our study, syrinxes had completely or significantly regressed following PFDD without additional surgeries in seven of a total eight patients; one patient had an unchanged syrinx cavity and presented with chronic symptoms for more than five years with unchanged surgical outcome.

Tubbs et al. figured out the differences in descent tonsillar ectopia between sagittal and coronal images and found 48 asymmetric ectopic tonsils on coronal images. Maximal left and right herniated tonsils were measured at 20.9 mm and 17.4 mm, respectively. The descents on midsagittal images were measured between 5 mm and 27.4 mm. On coronal images, 19 patients have one of the tonsils that were 3 mm below although each was 3 mm on the sagittal images. They concluded that CM1 had an asymmetrical tonsillar ectopia and the sagittal images overestimate the ectopic cerebellar tonsils in patients with CM1 [17]. In our study, tonsillar ectopia was calculated based on midsagittal MRI in 25 patients. Since the coronal MRI was added later, we had measured both coronal and midsagittal tonsillar ectopia in 17 patients. Though not all patients had descent below 5 mm on coronal images, the differences between the mean of pre -and postoperative images were statistically significant.

Since the opisthion is removed after PFDD in patients with CM1, coronal MRI can provide more accurate information at postoperative follow-up. Therefore, in patients with CM1 coronal MRIs should also be taken. In our study, syrinxes had regressed following PFDD without additional surgeries in 7 of 8 patients. The syrinx had regressed in three patients completely. Therefore, the PFDD is an effective surgical method in CM1 patients with or without syringomyelia [1].

The study suffers from a few limitations: 1) small sample size, 2) the retrospective nature, and 3) a single institution’s experience. Further larger sample-sized randomized prospective studies with a long follow-up period are mandatory.

## Conclusions

Based on our findings, PFDD is sufficient to relieve most of the major symptoms in patients with CM1 and resolve the syrinx cavity without additional surgical interventions. The CCOS is useful in the assessment of clinical outcomes. A reliable radiological evaluation should be performed on midsagittal and coronal images of MRIs.
